# Effect of an interprofessional care concept on the hospitalization of nursing home residents: study protocol for a cluster-randomized controlled trial

**DOI:** 10.1186/s13063-020-04325-y

**Published:** 2020-05-18

**Authors:** Alexandra Piotrowski, Martha Meyer, Iris Burkholder, Dagmar Renaud, Markus Alexander Müller, Thorsten Lehr, Sonja Laag, Joachim Meiser, Lisa Manderscheid, Juliane Köberlein-Neu

**Affiliations:** 1 University of Wuppertal, Wuppertal, Germany; 2 University of Applied Sciences Saarbrücken, Saarbrücken, Germany; 3 Saarland University, Saarbrücken, Germany; 4 BARMER, Wuppertal, Germany; 5 Saarland Association of Statutory Health Insurance Physicians, Saarbrücken, Germany

**Keywords:** Long-term care, Nursing home, Interprofessional care, Primary care, Cluster randomization, Collaboration, Complex intervention, Quality of life, Medication safety, Patient safety

## Abstract

**Background:**

The rising number of nursing home (NH) residents and their increasingly complex treatment needs pose a challenge to the German health care system. In Germany, there is no specialized geriatric medical care for NH residents. Nursing staff and general practitioners (GPs) in particular have to compensate for the additional demand, which is compounded by organizational and structural hurdles. As a result, avoidable emergency calls and hospital admissions occur.

In the SaarPHIR project (*Saarländische PflegeHeimversorgung Integriert Regelhaft*), a complex intervention focusing on a medical care concept was developed in a participatory practice-based approach involving NH representatives and GPs. The complex intervention addresses the collaboration between nurses and GPs and aims to help restructure and optimize the existing daily care routine. It is expected to improve the medical care of geriatric patients in NHs and reduce stressful, costly hospital admissions. The intervention was pilot-tested during the first 12 months of the project. In the present study, its effectiveness, cost-effectiveness, and safety will be evaluated.

**Methods:**

The study is a cluster-randomized controlled trial, comparing an intervention group with a control group. The intervention includes a concept of interprofessional collaboration, in which GPs group into regional cooperating teams. Teams are encouraged to cooperate more closely with NH staff and to provide on-call schedules, pre-weekend visits, joint team meetings, joint documentation, and improved medication safety. At least 32 NHs in Saarland, Germany (with at least 50 residents each) will be included and monitored for 12 months. The primary endpoint is hospitalization. Secondary endpoints are quality of life, quality of care, and medication safety. The control group receives treatment as usual. Process evaluation and health economic evaluation accompany the study. The data set contains claims data from German statutory health insurance companies as well as primary data. Analysis will be conducted using a generalized linear mixed model.

**Conclusion:**

A reduction in hospital admissions of NH residents and relevant changes in secondary endpoints are expected. In turn, these will have a positive impact on the economic assessment.

**Trial registration:**

German Clinical Trials Register: DRKS00017129. Registered on 23 April 2019. https://www.drks.de/drks_web/setLocale_EN.do.

## Introduction

One in four people in need of long-term care in Germany lives in a nursing home (NH) [1]. When compared to community-dwelling patients, NH residents are older and show greater limitations in activities of daily living and their health status. Considering the aging German population, this development is most likely to continue over future decades [1, 2]. These findings are supported by studies that show an increasing proportion of NH residents with multimorbidity and a high death rate during their first 12 months in residential care [3–5]. In addition, NH residents have a high degree of psychological and cognitive impairment [5]. A further critical consequence is that they have difficulties in following therapy instructions [6], which can lead to adverse drug events (ADEs) that are widely shown to be potentially avoidable [7, 8].

All this considered, it is becoming increasingly challenging to provide adequate medical care to NH residents [5, 6]. Whereas other countries (i.e., the Netherlands or France) provide specialized geriatric medical care for NH residents [9], in Germany this care is mainly carried out by general practitioners (GPs) in addition to their daily practice routine [9]. In this context, a German Health Technology Assessment (HTA) attests to an underuse and misuse of medical services due to a lack of interprofessional collaboration as well as poor documentation [4].

Besides that, medical care in NHs is not standardized [10]. Nursing staff report logistical and communicative difficulties with GPs, such as limited availability of GPs, challenging coordination, and insufficient documentation [11].

A few reliable contact persons, a trusting environment, fixed consultation hours, and regular visits are mentioned as promoting factors to overcome the described difficulties in NHs and foster successful collaboration [10].

The political response to this situation was the introduction of collaboration agreements between GPs and NHs in the German Social Insurance Code (*Sozialgesetzbuch [SGB] V*; in particular, agreements based on § 119b SGB V) in 2008. Over the following years, the law was amended several times until 2019, when a mandatory regulation on cooperation among GPs and NHs was added [12]. Furthermore, specific billing codes for GPs were introduced in 2016 [13]. However, NHs are still experiencing difficulties with implementation, as they have to negotiate independently, which means dealing with a lot of bureaucracy [12]. So far, the new regulations have not led to the desired success, namely a more efficient distribution of resources [14].

As before, potentially avoidable hospitalizations (PAHs) occur to a great extent [12, 15]. They are defined as events that can be handled in ambulatory care and do not necessarily have to be treated in hospital. These cases fall under the category of ambulatory care sensitive conditions (ACSC) (e.g., pneumonia, dehydration) [16]. A cross-sectional study on utilization patterns following the ACSC approach shows a significantly higher proportion of PAH among NH residents (27%) compared to people living at home (15%) [15].

At present, there are several projects in Germany which have recognized the limitations of the prevailing political approach and therefore aim to improve medical care in NHs and reduce PAH. In those projects, different priorities are set. Overall, interprofessional collaboration and its optimization is an important factor [17–19]. One project (Homern) deals with the reasons for hospital admissions and visits to the emergency room [20]. Another project (Study in Bavarian Nursing Homes) evaluates the prerequisites for successful collaboration between NHs and physicians [10, 21]. A third project (Careplus) aims at approaching a new concept of collaboration [18], a further project (CoCare) involves a technical solution [17], and one last project (interprof) provides a comprehensive science-based approach [19].

The project SaarPHIR (*Saarländische PflegeHeimversorgung Integriert Regelhaft*) addresses the solution through a practice-based approach, which was developed in a structured process in cooperation with GPs and NHs. This project also involves all German statutory health insurance (SHI) companies with clients in the study region, the Saarland Association of Statutory Health Insurance Physicians (German: *Kassenärztliche Vereinigung Saarland*), and the Saarland Association of Care (German: *Saarländische Pflegegesellschaft*). The Saarland Association of Care is the umbrella organization of various associations operating NHs in Saarland and thus functions as a valid representative of care issues.

### Objectives

SaarPHIR follows the hypothesis that residents of a long-term care facility benefit from structuralized processes at the interface between residential care and GPs. The main objective of the study is to investigate whether the developed and pilot-tested intervention can result in a reduction of hospitalization when compared to usual care. The primary outcome is hospitalization of the participating NH’s residents 12 months after baseline.

A further aim is to investigate whether medication safety, quality of life, and quality of care will improve. Therefore, the following secondary objectives will be evaluated at 12 months after baseline:

- Hospitalization specified via ACSC
- Hospital admissions from the residents’ point of view (based on data from resident files)
- Residents‘quality of life (Quality of Life in Alzheimer’s Disease [QoL-AD])
- Medication safety checks: number, scope, and performance
- Compliance with rules for documentation in terms of medication safety checks: performance and completeness of resident files; accessibility of information for NH staff, GPs, and other staff; integration into the care process
- Drug supply and use in emergency situations
- Compliance with rules for documentation in terms of screenings and assessments (e.g., geriatric screening, nutrition, and hydration): performance and completeness of resident files; accessibility of information for NH staff, GPs, and other staff; integration into the care process
- Compliance with rules for documentation in terms of hospital admissions: performance and completeness of resident files; accessibility of information for NH staff, GPs, and other staff; integration into the care process.

A health economic evaluation will be conducted alongside the study. In addition, a process evaluation will identify the barriers and facilitating factors of implementation, taking into account both perspectives, that of physicians and that of NHs. Secondly, the participating NHs are asked to share their experiences of the intervention. The process evaluation investigates the underlying mechanisms of the intervention in relation to the context and aims at interpreting summative results considering the impact of the intervention.

## Methods

### Study design

In order to assess whether the stated objectives have been attained by the SaarPHIR intervention, a prospective, pragmatic, cluster-randomized controlled trial (c-RCT) using two parallel groups with 1:1 randomization will be conducted.

**Fig. 1:**
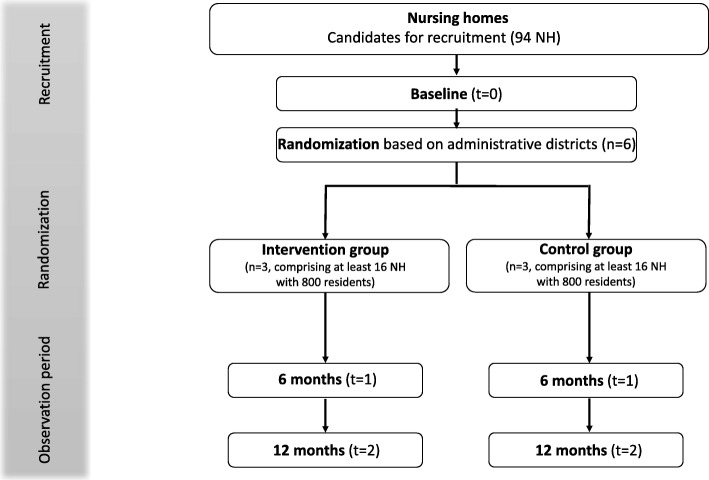
Flowchart of cluster-randomized controlled trial

The cluster level of randomization will be administrative districts (German: *Landkreise*) in Saarland. The reason for choosing this method was to avoid spillover effects, which are more likely to occur when analyzing at the NH level, because GPs are allowed to be contracted to more than one NH. Three districts will be randomly assigned to the intervention group, and three further districts to the control group. A minimum of 32 NHs is needed for the identification of significant effects. This means each group will comprise at least 16 NHs, each NH contributing at least 50 residents. Due to the intervention type, neither GPs and their patients nor participating NHs nor the study team will be blinded to treatment allocation. The observation period will be 12 months, with three points of data collection: at baseline (t_0_) and at 6 (t_1_) and 12 months (t_2_) after baseline (Fig. 1).

### Setting and trial population

#### Requirements for NHs

The study will be performed in (at least) 32 NHs in six administrative districts of Saarland (Regionalverband Saarbrücken, Saarpfalz-Kreis, Neunkirchen, St. Wendel, Merzig-Wadern, Saarlouis). Included will be NHs with at least 50 residents; each participating institution must give their consent before randomization. In the intervention group, the collaboration of the nursing facility with the participating physicians will be supported by a collaboration agreement (according to § 119b Abs. 2 SGB V).

#### Requirements for physicians

Interested physicians may participate in the study if they are members of the Saarland Association of Statutory Health Insurance Physicians and they work in a general practice located in Saarland. In addition to their own consent to participate in SaarPHIR, they must provide medical care to residents in at least one of the attending NHs. For GPs from the intervention group, it is additionally required to approve a collaboration agreement in accordance with § 119b paragraph 2 SGB V for the “promotion of cooperative and coordinated medical and nursing care in residential care facilities” (Annex 27 BMV-Ä).

Ideally, GPs ought not to treat patients from more than one district, to avoid spillover effects. The reimbursement structure of participating GPs is intended to support this. However, care that spans more than one district is not an exclusion criterion. During the evaluation, any overlaps, i.e., doctors who care for residents in both the intervention and the control groups, are identified and balanced in the statistical model using corresponding control variables.

#### Resident inclusion criteria

All residents living in residential care facilities in the six districts of Saarland will be included (base case analysis), as long as they are insured in the SHI system and classified by a level of care dependency (German: *Pflegegrad*) by their health insurance.

#### Recruitment and registration of NHs and physicians

A press meeting introducing SaarPHIR will aim to support the recruitment of NHs and physicians. Additionally, the Saarland Association of Statutory Health Insurance Physicians ( *Kassenärztliche Vereinigung Saarland*) will host three separate information events to inform physicians about the SaarPHIR project and the intervention.

After the information events, follow-up discussions will be offered in particular regions, explaining the intervention and its implementation in detail. Upon request, further on-site meetings in NHs are possible at any time, and telephone support is available. The target is to recruit at least 32 Saarland residential care facilities, so that approximately 1600 residents will be included for analysis.

#### Recruitment and registration of residents

Residents who meet the inclusion criteria will be informed and registered in their respective NHs. Participation in SaarPHIR is voluntary.

#### Written consent of the participating parties

For residents, participation forms including consent to the use of claims data are filled out in the NHs and provided to the health insurance companies. On this basis, the health insurance companies generate their claims data set.

For residents, NH staff, and GPs, consent forms for primary data collection are filled out in the NHs and remain there.

In both cases the evaluators do not have access to person-identifying data. Data collectors are not involved in data entry and evaluation. Model consent forms can be requested from the authors.

### Randomization and allocation concealment

Three districts (third cluster level) will be randomly assigned to the intervention group, and three further districts to the control group (1:1 allocation). Covariate-constrained randomization with data on hospitalization from the SHI will be used to achieve balanced study arms [22]. As a result, each group will comprise at least 16 NHs (second cluster level) and 800 residents (32 NHs and 1600 residents in total) (first cluster level).

The randomization list will be computer-generated by an independent researcher who will be blinded to the districts. Allocation results will be reported to the project coordinator and communicated in an on-site information meeting in the respective institutions. Due to the type of intervention, neither GPs and their patients nor participating NHs nor the majority of the study team will be blinded to treatment allocation. The only exception is the researcher who will perform the statistical analysis of the primary outcome parameter; he or she will be blinded to the treatment allocation.

### Intervention

**Fig. 2:**
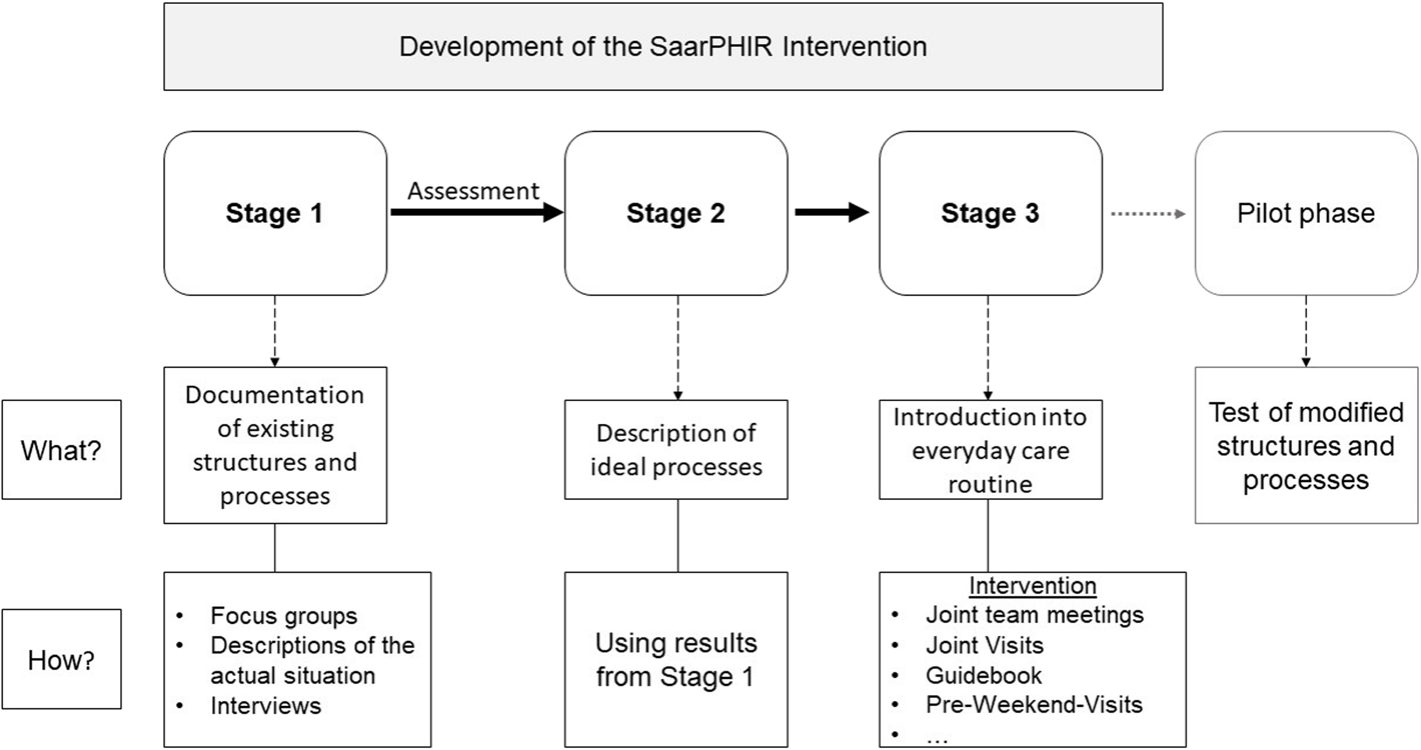
Development of the SaarPHIR intervention

International publications suggest that residents benefit from improved processes and continuous care [23, 24], especially in terms of reducing hospitalization [24, 25], while at the same time, the satisfaction of employees with their daily work and their role in the health care system improves [23]. In order to align with this concept, the intervention was developed in three major stages or steps (Fig. 2):

1. Systematic documentation of existing structures and processes
2. Definition of ideal processes, based on the information of step 1
3. Introduction into everyday care routine.

The described development process resulted in an intervention comprising various components and processes, as described in the following subsections.

#### Reorganization of physicians

Participating physicians will be grouped into regional teams (medical care teams). Each team chooses a representative to plan on-call duties and pre-weekend visits. Planning is carried out on the basis of the entire team and thus reduces the organizational effort of the individual team members. The joint planning implies that, in exceptional cases, patients might be treated by doctors other than their family doctor, for example, as part of the on-call duty or the pre-weekend visit. This way, a quick response can be made in critical situations. To help with this, it is important that all residents (if possible) make written arrangements for emergencies or complete an advanced directive (or their own document for emergency situations) to be kept in the residents’ files. Regardless of these changes, the freedom to choose a doctor remains unaffected.

#### Team meetings

Team meetings will be held regularly (at least four times a year) between the medical care team and the responsible nursing staff in order to exchange information, provide further joint training, discuss cases, and jointly solve problems in crisis situations. The nursing facilities will designate a coordinating nurse (plus deputy) as the contact person for collaboration with the medical care team.

#### Extended on-call duty

A GP from the care team will be on duty from Monday to Friday. This ensures that, in addition to the regular consultation times, at least one GP from the care team is available from 6 pm to 9 pm. This timeframe was considered particularly important by the initiators of the project in order to avoid hospitalization. A duty roster for each quarter will be drawn up 4 weeks in advance and provided to the NH staff.

#### Pre-weekend visit

The medical care team will ensure a regular pre-weekend visit in the form of a Friday afternoon or Saturday visit. These visits will follow certain organizational rules. First, NH staff will list who is to be visited and compile all relevant information. Only acute cases are to be covered. GPs will be informed about the cases prior to their visit. The pre-weekend visit is not to replace any physician’s regular visit during the week.

#### Regular screenings and assessments

Part of the intervention is the regular performance of screenings and assessments. Mandatory on admission to the NH, and once a year thereafter, is a basic geriatric screening (Lachs screening) and fall prevention. Further assessments (in terms of nutrition/hydration, dementia, depression/anxiety, and activities of daily living or mobility) can be carried out if relevant. Each screening must be documented in the resident file, and the completed form must be attached. An overview of all screening and assessment instruments as well as their templates can be found in the guidebook (see the subsection on Implementation strategy).

#### Coordinating nurse

As the medical care teams are each to be chaired by a coordinating GP, on the side of the NH one designated nurse will also be entrusted with a coordinating function. Among other things, coordinating nurses will be required to take part in joint team meetings and the organization of pre-weekend visits. In order to support the prevailing NH staff structures, a part-time position (25%) for each facility participating in the intervention will be financed by SaarPHIR.

#### Implementation strategy

Information events will be offered (by the Saarland Association of Statutory Health Insurance Physicians and the Association of Care) in particular regions, explaining all details regarding the intervention and its implementation. Upon request, further on-site meetings in NHs will be possible at any time, and telephone support will be available.

A guidebook summarizing the components of the SaarPHIR intervention, providing information and templates of the relevant screening and assessment instruments, is intended to support the teams and function as a reference manual.

#### Control group

The control group will receive treatment as usual. Each resident is to be treated by his or her GP. This takes place as part of the GP’s daily practice routine. There are no contractual agreements between the NH and the GP and no additional billing codes. The organization of medical and nursing care is to be handled by current NH staff. There will not be a coordinating nurse.

### Outcome measures

Three measurement points are planned: baseline assessment (t_0_) and two measurements (t_1_, t_2_) after 6 and 12 months (see Table 1).

**Table 1:** Measurements used in SaarPHIR trial

Level	Measure point	Assessment	Measure
Institutional level	t_0_ baseline t_1_ 6 months t_2_ 12 months	Characteristics/organizational structure of NH, Resident structure Characteristics/organizational structure of GP practice, Contextual factors, Intervention status, Reasons for drop-out, Interprofessional communication, Barriers and facilitators of implementation	NH accounting system GP accounting system Questionnaire Status form (self-developed) [26, 27] Interviews Questionnaire [28, 29] Focus groups, interviews
	t_2_ 12 months	Organizational change	Organizational readiness for implementing change (ORIC) [30]
Resident level	t_0_ baseline t_1_ 6 months t_2_ 12 months	Sociodemographic data, Diagnosis data, Hospitalization, Medication data, Billing data, Cognition, Quality of life, Screenings/assessments, Documented hospitalization, Medication safety checks/documentation/use	Claims data Claims data Claims data Claims data Claims data DSS [31] QoL-AD [32] Resident file Resident file Resident file

#### Primary outcome

The primary outcome in SaarPHIR is the hospitalization of residents within 12 months from the start of the study. First, as well as recurrent, hospitalizations will be taken into account. The analysis will be primarily based on claims data.

#### Secondary outcomes

The number of avoidable hospital admissions, following the ambulatory care sensitive conditions (ACSC) approach, will be determined. The reference used is a collection of Germany-specific diagnoses [16]. Because the German version does not only cover diagnoses specific to NH residents, and therefore does not explicitly address the SaarPHIR target group, a sensitivity analysis will be conducted with a reduced number of ACSC diagnoses [33].

A sample of at least 10% of the residents in interventional care will be drawn to map the residents’ perspective. For this, hospitalization data from their resident files will be examined at t_0_, t_1_, and t_2_.

The same 10% sample of residents will be asked about their quality of life, using Edelman’s adaption of the Quality of Life in Alzheimer’s Disease scale (QoL-AD) [32] at t_1_ and t_2_. This particular adaption allows both self-assessment of the residents or assessment by proxy (caregiver or relative). The Dementia Screening Scale (DSS) [31] will be used in advance to assess cognitive ability. If the result is 4 or higher, the resident will complete the questionnaire himself/herself; otherwise, the proxy will be used. Studies indicate that the QoL-AD is able to provide useful information with a DSS score > 4 [31, 34].

Again, the same 10% sample of residents in interventional care will be reviewed to evaluate the performance of medication safety checks and to measure the quality of its documentation (at t_1_ and t_2_). Data on drug supply, emergency drug usage, availability of resident-specific information, and drug handling complete the data set. This enables the degree of implementation of medication safety tools to be directly compared to variations in results among different care facilities. This includes an analysis of the handling and medical documentation of on-demand medications by both physicians and caregivers (see Table 1).

Files of the aforementioned 10% sample will be used to examine compliance with the rules for documentation in terms of screenings and assessments (e.g., geriatric screening, nutrition, and hydration) and in terms of hospital admissions (at t_1_ and t_2_). The standard of documentation and completeness of the files, accessibility of information for NH staff, GPs, and other staff, as well as integration into the care process will be assessed.

#### Health economic evaluation

Using the incremental cost-effectiveness ratio (ICER), the cost-effectiveness of the SaarPHIR intervention will be estimated. The ICER indicates the difference between the average costs of the control and intervention groups and the incremental effectiveness between both groups [35]. The effectiveness will be represented by a reduction in hospitalization [35]. In order to avoid double counting of costs, the costs of health services in the denominator of the ICER will be corrected for the costs incurred by the use of inpatient services. Furthermore, a budget impact analysis (BIA) will be conducted in compliance with the Principles of Good Practice for Budget Impact Analysis reported by the International Society for Pharmacoeconomics and Outcomes Research (ISPOR) in order to estimate the impact of the SaarPHIR intervention on the respective budgets of the parties involved in SaarPHIR [36, 37].

#### Contextual factors

Data on the characteristics and organizational structures of the NHs [38] and general practices, the status of the intervention, and the quality of the collaboration [21] between NHs and physicians will be surveyed. In addition, the resident structure (drop-out rate and reasons), as well as pre-weekend visits carried out, will be documented on a weekly basis. The nursing staff will be encouraged to respond to interviews and questionnaires on behalf of their facility.

#### Process evaluation

A comprehensive process evaluation from the beginning to the end of the SaarPHIR study is necessary to understand the underlying mechanisms of the intervention and the contextual influences, in order to ensure the generalizability of the study results as well as to improve future implementations. Process evaluation outcomes will be collected according to a framework for cluster-randomized trials of complex interventions [39].

A mixed-methods approach will be used to collect data alongside the c-RCT. Every 3 months, NH representatives and physicians will document the progress of the intervention. Furthermore, the process evaluation addresses the recruitment procedure and reasons for non-participation, as well as contextual factors and organization-related questions (see Table 1).

### Data collection and management

#### General aspects

Secondary data are to be provided by the health insurance companies. Primary data will be collected by questionnaires or interviews. The questionnaires are to be handed out in paper form. Some of the questionnaires are proxy surveys. The original questionnaires will be sent to the University of Wuppertal, where they will be scanned using an automatic capture system (Teleform TF V16, Electric Paper Informationssysteme GmbH) and added to the databank. Interviews will be transcribed and entered to the databank as well.

A project-specific data security concept has been developed and will be implemented to ensure a high level of security of all collected data. The concept takes into account current European and German law and thus ensures the integrity and security of the collected data. All data will be pseudonymized in a multistep process, involving an external data trustee. Access to the data will be granted only to the researches involved in the evaluation.

#### Data collection based on claims data

Calculations of the primary and secondary endpoints as well as the sensitivity analysis will mainly be carried out with SHI claims data. Generally, claims data describe the billable services of insured patients; thus, they reflect the patients’ interaction with the health care system. Data for all potentially eligible patients will be collected from the relevant insurers’ claims data sets. The sources of the required claims data refer to several categories (§ 284 paragraph 1 SGB V): inpatient (§ 301 SGB V), outpatient (§ 295 SGB V), outpatient surgery (§ 115b SGB V/§ 116b SGB V), long-term nursing care (SGB XI), pharmacy (§ 300 SGB V/§ 302), master data, and Disease Management Program (DMP) billing.

#### Data collection based on primary data and other administrative data

For the evaluation of structure and process quality as well as medication safety, primary data and further administrative data will be collected in addition to the described claims data analysis (see the subsection Outcome measures):

- Quantitative data in terms of structure and contextual factors for coordinating physicians and nursing staff (t_0_, t_2_)
- Quantitative data in terms of intervention fidelity and process changes for coordinating physicians and nursing staff (t_0_, follow-up every 3 months)
- Communication questionnaires for coordinating physicians and NH staff (t_0_, t_1_, t_2_)
- Quality of life questionnaire for residents: QoL-AD (t_0_, t_1_, t_2_)
- Data from the resident files in order to assess the quality of care (t_0_, t_1_, t_2_)
- Questionnaires on medication safety for NH staff (t_0_, t_1_, t_2_)
- Focus groups with physicians and nursing staff (coordinating persons) (t_1_, t_2_)
- Qualitative interviews with physicians (t_1_)
- Qualitative interviews with NH staff (t_1_)
- Qualitative interviews with physicians and NHs about reasons for non-participation (after recruitment).

### Statistical analysis

The researcher who will perform the statistical analysis of the primary outcome parameters will not be involved in the conduction of the study. Furthermore, this researcher will be blinded to the group allocation of NHs and residents. Analysis of the primary outcome is based on SHI claims data for the years 2018 to 2020. The analysis level for the primary outcome measure, i.e., hospitalization rate, will be the second cluster level (care facilities, *n* = 16 per group, 32 in total). The primary statistical analysis will be conducted as an intention-to-treat analysis, using first, as well as recurrent, hospitalizations as a dependent variable in a generalized linear mixed model, with the intervention as a fixed effect and clusters as a random effect. A constant multiplicative dispersion parameter will be estimated to account for the dependence of recurrent events. An imbalance of baseline characteristics can be expected, which cannot be compensated by randomization. For this reason, the intervention effect will be adjusted for the characteristics of the NHs, e.g., baseline hospitalization rate, as well as for characteristics of the participants, e.g., gender and age. The intervention effect will be tested with α = 5% [40]. Furthermore, cluster-adjusted 95% confidence intervals will be calculated. The sensitivity analyses will include a per-protocol analysis as well as the analyses of subgroups using a generalized linear mixed model. Secondary outcomes will be analyzed explanatively with linear, generalized linear mixed or time-to-event models and will be based on claims data as well as collected primary data and data from the resident’s file.

Quantitative data of the process evaluation will be analyzed descriptively, graphically, and numerically. Process changes are to be analyzed via process observations.

#### Qualitative data

Qualitative data from interviews and focus groups will be audio-recorded if consent has been given. They will then be transcribed and coded by two researchers using qualitative content analysis.

#### Study population

The study sample is generated on the first cluster level. Therefore, all insured residents living in an NH in Saarland at the beginning of the study will be included (closed cohort design). This means all residents will be included, not just those attending SaarPHIR via selective agreement. By doing this, calculations will be conducted on a population level, which, in turn, helps to avoid selection effects. Residents are under observation until the end of the study, leaving the NH, or death.

All residents living in participating NHs in Saarland will be included in the assessment of the primary outcome (base case analysis, population level) as long as they are insured via SHI and classified by a level of care dependency by their health insurance (*Pflegegrad*). Furthermore, these residents will also be included in the analysis of secondary outcomes. Analyses of secondary outcomes based on primary data (e.g., quality of life) may only be carried out on residents receiving the intervention (since only these persons give their written consent). The contractual basis for the medical care of SaarPHIR residents is a selective agreement with their respective health insurance companies.

#### Sample size

In order to analyze the primary outcome, hospitalization data will be collected for 12 months and adjusted to data from the previous year (baseline). For an effect size of 0.6, assuming a significance level of 5% (α = 0.05) and a power of 80% (β = 0.20), at least 32 facilities with 50 residents each (assuming 30 person-days at risk per resident) are required (calculated with the R package ClusterPower, hayer.power.poisson [41]). Furthermore, a between-cluster variance (BCV) of 0.10 has been calculated. Instead of the intraclass correlation coefficient (ICC), the package ClusterPower refers to the BCV as a measure to account for the degree of clustering. The BCV is equivalent to the ICC and was used by Hayes and Bennett due to its better comprehensibility [42].

Secondary endpoints will be evaluated following exploratory approaches; therefore, a sample size calculation is not needed.

#### Sensitivity analysis

Sensitivity analyses are an essential part of every scientific evaluation and an important basis for decision-making [43]. The calculations refer to the same data basis as the primary analysis. The sensitivity analyses are therefore an extension of the primary analyses, for example, with a specific subgroup analysis. Data from 2018 to 2020 will be used for the following populations:

1. Insured residents who live in participating NHs at the start of the study *or* during the course of the study (open cohort design)
2. An intervention group consisting of *only participating insured residents* (selective agreement, open cohort design without regard to a potential selection bias)
3. All insured residents in Saarland.

For study population 3, both the “open cohort design” and the “closed cohort design” will be used. In addition, the primary endpoint for the intervention group is validated with data from the resident files, to ensure that health claims data cover the entire range of hospital (re-)admissions.

### Duration of the project

SaarPHIR started in April 2018 and should be completed by March 2021. The c-RCT started in May 2019. Data will be archived for 10 years after study completion.

### Ethical and legal considerations

This project has received ethical approval from the Ethics committee of the Saarland Medical Association, No. 56/18.

### Dissemination policy

Study results will be published in a peer-reviewed, MEDLINE-listed journal.

A German website (https://saarphir.kvsaarland.de/) was created to provide study materials and to publish study results at a later stage.

## Discussion

The present study investigates the effect of an interprofessional collaboration concept in nursing homes on the hospitalization of residents. The concept addresses nursing home staff and physicians. The method used is a cluster-randomized controlled trial. The intervention group is treated with the new concept; the control group receives treatment as usual. The database consists largely of statutory health insurance claims data. In addition, primary data are collected.

Since changes on an organizational and structural level are being addressed, blinding is not possible. A total of 32 nursing homes with 1600 patients will be examined. Some primary data collections (e.g., quality of life) are carried out on a 10% sample in order to keep the burden on the respondents as low as possible. Apart from this, however, a large database is ensured, so that the transferability of the results can be considered high. This is also supported by the mixed-methods approach.

A reduction in hospital admissions (hospitalization) of the residents and relevant changes in secondary endpoints are expected. In turn, these will have a positive impact on the economic assessment. International publications support these assumptions [23, 25]. The process evaluation will also help us to understand how the success of the implementation process can be facilitated. It is expected that, based on the results of the study, recommendations can be made for the care provided in German nursing homes.

### Trial status

The trial is at Version 1, February 2019.

Ethics committee approval, No. 56/18, was provided by the Ethics committee of the Saarland Medical Association, 04.03.2019. The primary recruitment phase of the trial began on 1.05.2019 (protocol version 1, 05.02.2019). The end of the trial is scheduled for 31.04.2020. Extension by probably 6 months is planned.

## Data Availability

Not applicable.

## Abbreviations

ACSC: Ambulatory care sensitive conditions
BCV: Between-cluster variance
BIA: Budget impact analysis
BMV-Ä: Bundesmantelvertrag-Ärzte (Federal Master Treaty for Medical Practitioners)
c-RCT: Cluster-randomized controlled trial
DMP: Disease Management Program
DSS: Dementia Screening Scale
GP: General practitioner
HTA: Health Technology Assessment
ICC: Intraclass correlation coefficient
ICER: Incremental cost-effectiveness ratio
ISPOR: International Society for Pharmacoeconomics and Outcomes Research
NH: Nursing home
PAH: Potentially avoidable hospitalization
QoL-AD: Quality of Life in Alzheimer’s Disease
SGB: Sozialgesetzbuch (German Social Insurance Code)
SHI: Statutory health insurance
